# Species-dependent variation of the gut bacterial communities across *Trypanosoma cruzi* insect vectors

**DOI:** 10.1371/journal.pone.0240916

**Published:** 2020-11-12

**Authors:** Luisa M. Arias-Giraldo, Marina Muñoz, Carolina Hernández, Giovanny Herrera, Natalia Velásquez-Ortiz, Omar Cantillo-Barraza, Plutarco Urbano, Juan David Ramírez

**Affiliations:** 1 Grupo de Investigaciones Microbiológicas-UR (GIMUR), Departamento de Biología, Facultad de Ciencias Naturales, Universidad del Rosario, Bogotá, Colombia; 2 Grupo de Biología y Control de Enfermedades Infecciosas, Universidad de Antioquia, Medellín, Colombia; 3 Grupo de Investigaciones Biológicas de la Orinoquia, Fundación Universidad del Trópico Americano (Unitropico), Yopal, Colombia; University of Minnesota Twin Cities, UNITED STATES

## Abstract

Triatomines (Hemiptera: Reduviidae) are the insect vectors of *Trypanosoma cruzi*, the causative agent of Chagas disease. The gut bacterial communities affect the development of *T*. *cruzi* inside the vector, making the characterization of its composition important in the understanding of infection development. We collected 54 triatomine bugs corresponding to four genera in different departments of Colombia. DNA extraction and PCR were performed to evaluate *T*. *cruzi* presence and to determine the discrete typing unit (DTU) of the parasite. PCR products of the bacterial 16S rRNA gene were pooled and sequenced. Resulting reads were denoised and QIIME 2 was used for the identification of amplicon sequence variants (ASVs). Diversity (alpha and beta diversity) and richness analyses, Circos plots, and principal component analysis (PCA) were also performed. The overall *T*. *cruzi* infection frequency was 75.9%, with TcI being the predominant DTU. Approximately 500,000 sequences were analyzed and 27 bacterial phyla were identified. The most abundant phyla were Proteobacteria (33.9%), Actinobacteria (32.4%), Firmicutes (19.6%), and Bacteroidetes (7.6%), which together accounted for over 90% of the gut communities identified in this study. Genera were identified for these main bacterial phyla, revealing the presence of important bacteria such as *Rhodococcus*, *Serratia*, and *Wolbachia*. The composition of bacterial phyla in the gut of the insects was significantly different between triatomine species, whereas no significant difference was seen between the state of *T*. *cruzi* infection. We suggest further investigation with the evaluation of additional variables and a larger sample size. To our knowledge, this study is the first characterization of the gut bacterial structure of the main triatomine genera in Colombia.

## Introduction

Triatomines (Hemiptera: Reduviidae) are the insect vectors of *Trypanosoma cruzi*, the causative agent of Chagas disease [1], a neglected tropical disease (NTD) that affects 21 countries in which the vector is distributed, including Colombia where the annual prevalence is around 2% [2], showing a heterogeneous geographical distribution mainly associated with conditions of poverty [3]. The parasite presents tremendous genetic diversity and has been divided into six discrete typing units (DTUs), from TcI to TcVI [4]. Upon entry into insects, the parasites settle in the gut where they replicate, differentiate (metacyclogenesis), and proliferate [5]. Therefore, the triatomine gut and its contents might determine the ability of the insects to acquire, maintain, replicate, and/or transmit *T*. *cruzi* [6–9].

Triatomines belong to the subfamily Triatominae, which is composed of approximately 152 extant species present in the Americas [10]. The main triatomine vectors in the Americas belong to the genera *Rhodnius*, *Triatoma*, and *Panstrongylus* [1,11]. In Colombia, 15 of the 24 reported species are naturally infected by *T*. *cruzi*, with *Rhodnius prolixus*, *Triatoma dimidiata*, and *Panstrongylus geniculatus* being the main vector species found throughout the country [12]. *Psammolestes*, despite being considered a genus associated with bird nests, was recently found to be naturally infected with *T*. *cruzi* [13].

The gut microbiota affects the development of *T*. *cruzi* inside the vectors [9,14–16]. The main biological variables that have been suggested to shape the gut bacterial structure of triatomines include triatomine species, feeding sources, and presence of *T*. *cruzi*. Numerous studies have found the gut bacterial communities to differ depending on triatomine species [7,17,18]. Regarding the feeding sources, Dumonteil et al. [19] found that the bacterial orders present in *T*. *dimidiata* changed when the feeding source was altered, although there have been reports that do not reach the same conclusion [20]. More importantly, the same study found that one triatomine is able to feed from multiple hosts, which suggests frequent host changes that can affect the transmission dynamics of *T*. *cruzi* and bacterial communities in the triatomine gut [19]. Díaz et al. [7] showed that the diversity of the gut microbiota increases when the parasite is present, which was supported in later studies [17,18], although other reports have found no links between these factors [20].

The gut bacterial communities of *Rhodnius*, *Triatoma*, and *Panstrongylus* have been shown to be composed of only one predominant phylum, Actinobacteria, [21], which was later identified simultaneously with Proteobacteria, Firmicutes, and Bacteroidetes [17,22]. Dumonteil et al. [19] found 23 bacterial orders present in *T*. *dimidiata*. Of these bacterial orders Bacillales, Actinomycetales, Enterobacteriales, and Burkholderiales were most abundant. More recently, a study by Kieran et al. [20] detected Actinobacteria, Bacteroidetes, Firmicutes, and Proteobacteria as the most abundant bacterial phyla in *R*. *pallescens*. Despite these studies, knowledge of this subject remains scarce given the limitations in terms of the countries in which this has been evaluated and the few triatomine species that have been analyzed. Moreover, to date, limited studies have been performed in Colombia, one of the countries with the highest incidence of Chagas disease [3].

In this study, we conducted the first characterization of the gut bacterial communities of the main triatomine genera in Colombia (*Panstrongylus*, *Rhodnius*, and *Triatoma*) and *Psammolestes*, which was included due to recent evidence of *T*. *cruzi* infection. We also compared the composition of bacterial communities among (i) insects naturally infected or not infected with *T*. *cruzi* and (ii) insects collected in different regions of the country.

## Materials and methods

### Insect sampling and dissection

We collected 54 triatomine bugs corresponding to four genera (12 *P*. *geniculatus*, 7 *Psammolestes arthuri*, 8 *R*. *pallescens* and 21 *R*. *prolixus*, and 3 *Triatoma maculata* and 3 *Triatoma venosa*) from 2012 to 2018 in different departments of Colombia (Arauca, Bolívar, Boyacá, Casanare, La Guajira, Magdalena, Meta, and Santander) (S1 Fig, S1 Table). All the insects used were adults. The insects were collected using different entomological surveillance techniques for each ecotope (domestic, peridomestic, and sylvatic), which have been described elsewhere [23]. Universidad del Rosario provided the field permit from ANLA (Autoridad Nacional de Licencias ambientales) 63257–2014. All collection was done on public land. Bugs were kept in 100% ethanol in Eppendorf tubes and stored at -20 °C until dissection. Dissection resulted in two tubes per insect: one containing the head, legs, and scutellum and one with the abdominal region of the insect. The majority of the abdominal regions dissected seemed to be engorged (i.e. the insect had recently fed upon collection). The abdominal region was washed three times with PCR ultra-pure water and used for further analyses.

### Detection and genotyping of *T*. *cruzi*

DNA was extracted using the DNeasy Blood & Tissue Kit (Qiagen, Germany) with minor modifications of the manufacturer’s protocol. DNA concentrations were determined with a NanoDrop ND-100 spectrophotometer (Thermo Fisher Scientific Inc., Massachusetts, USA).

The presence of *T*. *cruzi* was detected by qPCR with primers Cruzi1 (ASTCGGCTGATCGTTTTCGA), Cruzi2 (AATTCCTCCAAGCAGCGGATA), and probe Cruzi3 (CACACACTGGACACCAA), as described elsewhere [24,25]. The results were considered positive when the amplification exceeded the threshold of fluorescence of 0.01. For the insects with positive results by qPCR, *T*. *cruzi* was distinguished from *T*. *rangeli* to avoid any possible bias produced by this trypanosomatid. This differentiation was made by amplifying the DNA in the kinetoplast fragment using primers 121 (AAATAATGTACGGGKGAGATGCATGA) and 122 (GGTTCGATTGGGGTTGGTGTAATATA), as described elsewhere [26]. *T*. *rangeli* was not identified in any of the evaluated samples. For the insects identified as *T*. *cruzi*-positive by qPCR, the DTU of the parasite was identified using primers targeting the spliced-leader intergenic region of the mini-exon (SL-IR), as described by Hernández et al. [24]. This resulted in the discrimination of TcI and TcII-TcVI DTUs. TcI-positive samples were further categorized into TcIDom and TcISylv using primers 1AM (TGTGTGTGTATGTATGTG) and 1B (CGGAGCGGTGTGTGCAG), which also target the SL-IR, as described elsewhere [27].

### Identification of bacterial communities

Amplicon sequencing was performed by Novogene (Beijing, China). PCR products of the bacterial 16S rRNA gene were pooled for sequencing after independent library construction and 2 × 300 paired-end sequencing was performed on an Illumina HiSeq flow cell (Illumina, San Diego, USA). For the amplification of the V4 region, the universal primers 515F (5’-GTGCCAGCMGCCGCGGTAA-3’) and 806R (5’-GGACTACHVGGGTWTCTAAT-3’) were used, which amplify a sequence fragment of 390 bp. This particular region of 16S rRNA was chosen because it has been identified as one of the most accurate and includes archaea members [28]. The analyses of amplicon-based sequence data were performed using QIIME 2 software [29]. Initially, the raw reads obtained from Illumina paired end sequencing were cleaned by extracting the attached barcodes and primers, and removing the sequencing noise. Those sequences with incongruences in the barcode or without the correct primer sequence as well as short (< 200bp of lenght) and low quality (with a minimal average quality score of 25) reads were discarded.

Forward and reverse high quality reads were merged together to obtain the full denoised sequences by aligning using PyNAST [30]. Operational Taxonomic Units (OTUs) were then defined considering an identity of 97%. The taxonomic identification of OTUs was developed by comparison against Ribosomal Database Project (RDP) Classifier reference sequences [31] using the uclust consensus taxonomy assigner [32]. Bacteria detected with less than 3% abundance in the bacteriome of an individual triatomine were excluded from analysis. The number of reads obtained for each taxon in each sample was used as a proxy of its abundance in a given sample, as previously reported [20].

To assess diversity, we measured alpha and beta diversity by calculating the inverse Simpson diversity index and Bray-Curtis dissimilarity distance, respectively, by considering, as different sites for beta diversity analysis, (i) the geographical origin within the country (departments) where the insects were collected and (ii) the ecotopes to which the detected *T*. *cruzi* DTUs corresponded, which were defined as “Domestic”, “Sylvatic”, and “Domestic & Sylvatic” and corresponded to the characteristics of the place where the triatomine was collected.

We also conducted principal component analysis (PCA) with R, using the package “factoextra” to observe potential patterns among bacterial groups and triatomine species. Additionally, we calculated the Margalef richness index and constructed rarefaction curves to estimate species richness of our sampling. Additionally, we used the online tool Circos (http://circos.ca/) to graphically represent the relative abundance and distribution of bacteriome components in triatomines per species and per infection state for both cases [33].

### Statistical analyses

Variables such as triatomine species, ecotope (domestic, peridomestic, sylvatic), presence of *T*. *cruzi*, bacterial taxa comprising the gut bacteriome, and triatomine feeding sources were initially treated as categoric variables. The association between these variables was evaluated using Pearson’s chi-squared test. Odds ratio (ORs) and corresponding CI95% were calculated to measure the strength of association between the analyzed variables. Additionally, t-tests were performed with R 3.6.2 to evaluate differences in diversity between each bacterial phylum.

## Results

### Detection and genotyping of *T*. *cruzi*

The overall *T*. *cruzi* infection frequency was 75.9% (*n* = 41). The frequency of *T*. *cruzi* infection within each species was as follows: *P*. *geniculatus*, 83.3% (10/12); *Ps*. *arthuri*, 57.1% (4/7); and *R*. *prolixus*, 61.9% (13/21). In the cases of *R*. *pallescens*, *T*. *maculata*, and *T*. *venosa*, the frequency of infection was 100%.

Analysis of DTUs identified TcI and TcII-TcVI in 35 samples, with TcI being the predominant DTU (80%, *n* = 28). We also found six (17.1%) mixed infection cases (TcI and TcII-TcVI simultaneously), which corresponded to four *P*. *geniculatus*, one *Ps*. *arthuri*, and one *R*. *prolixus*. The only case of TcII-TcVI (2.8%) detected belonged to a *T*. *maculata* insect. Six cases (17.1%) were unrecognized (UR); in these samples DTUs, despite *T*. *cruzi*-positive results, could not be determined due to parasitic loads being too low to be typed. The UR samples found in this case corresponded to one *R*. *pallescens*, five *R*. *prolixus*, and one *T*. *maculata*. Among the positive samples for DTU TcI, six *P*. *geniculatus* (21.4%), three *Ps*. *arthuri* (10.7%), seven *R*. *pallescens* (25%), eight *R*. *prolixus* (28.6%), one *T*. *maculata* (3.6%), and three *T*. *venosa* (10.7%) were detected.

Among the samples identified as TcI, TcIDom and TcISylv were found. We detected nine (32.1%) TcIDom, of which two corresponded to *P*. *geniculatus*, one to *Ps*. *arthuri*, four to *R*. *pallescens*, one to *R*. *prolixus*, and one to *T*. *venosa*. Similarly, the four cases detected as TcISylv (14.3%) belonged to *R*. *prolixus* samples. We also detected six (21.4%) mixed infection cases, where both TcIDom and TcISylv were found simultaneously, composed of three *P*. *geniculatus*, two *Ps*. *arthuri*, and one *R*. *prolixus*. The nine UR cases detected (32.1%) corresponded to one *P*. *geniculatus*, three *R*. *pallescens*, two *R*. *prolixus*, one *T*. *maculata*, and two *T*. *venosa*.

### Identification of bacterial communities

**Fig 1:**
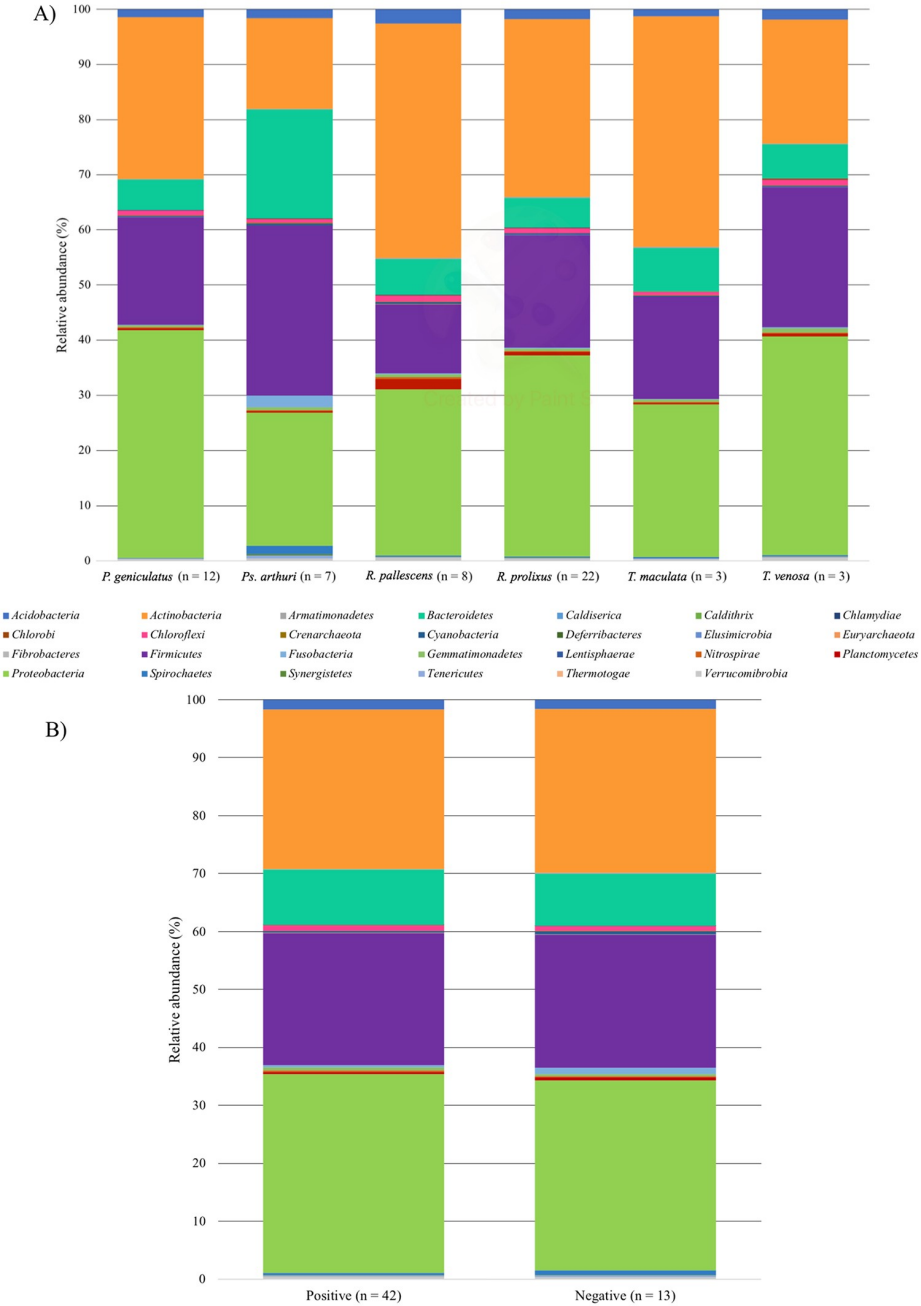
Relative abundance of bacterial phyla (A) in each triatomine species evaluated, and (B) depending on the presence or absence of *T*. *cruzi*. The number of samples from which the information was collected is shown below each bar.

Approximately 500,000 sequences were analyzed (with an average of 8900 reads per sample), and 27 bacterial phyla were identified (Fig 1, S2 Fig). Rarefaction curves indicated that a large proportion of the richness had been identified by our sampling method in this particular set of data (see S3 Fig). Phylum and genus were the taxonomic levels used in this study given that they were the most widely identified and, therefore, more reliable for posterior analyses. The most abundant phyla were Proteobacteria (33.9%), Actinobacteria (32.4%), Firmicutes (19.6%), and Bacteroidetes (7.6%), which together accounted for over 90% of the gut bacterial structure identified in this study. These four phyla were the main targets for the identification of bacterial genera, the results of which were: 14 Actinobacteria (S4a Fig), 18 Bacteroidetes (S4b Fig), 14 Firmicutes (S4c Fig), and 13 Proteobacteria (S4d Fig), which accounted for 59 bacterial genera. Among these genera, there were noteworthy cases identified like *Wolbachia* (73 reads, 0.15% of Proteobacteria reads), *Serratia* (230 reads, 0.47% of Proteobacteria reads), and *Rhodococcus* (126 reads, 0.18% of Actinobacteria reads) bacterial genera. It is important to mention that all of the *Wolbachia* reads were found in *R*. *prolixus* bugs, while *Serratia* was found mostly in *P*. *geniculatus* but also in *R*. *prolixus*, and *Rhodococcus* was present in *P*. *geniculatus*, *Ps*. *arthuri*, and *R*. *prolixus* (see S5 Fig). Particular attention was paid to the presence of these genera given previous studies suggesting that these bacteria could affect the *T*. *cruzi*-triatomine´s gut interaction [34,35]. Actinobacteria was the phylum in which the difference between triatomine species was most notable, especially in the case of *Ps*. *arthuri* (Fig 1A, S6 Fig). In regard to *T*. *cruzi* presence, there were no noticeable differences among samples (Fig 1B).

**Fig 2:**
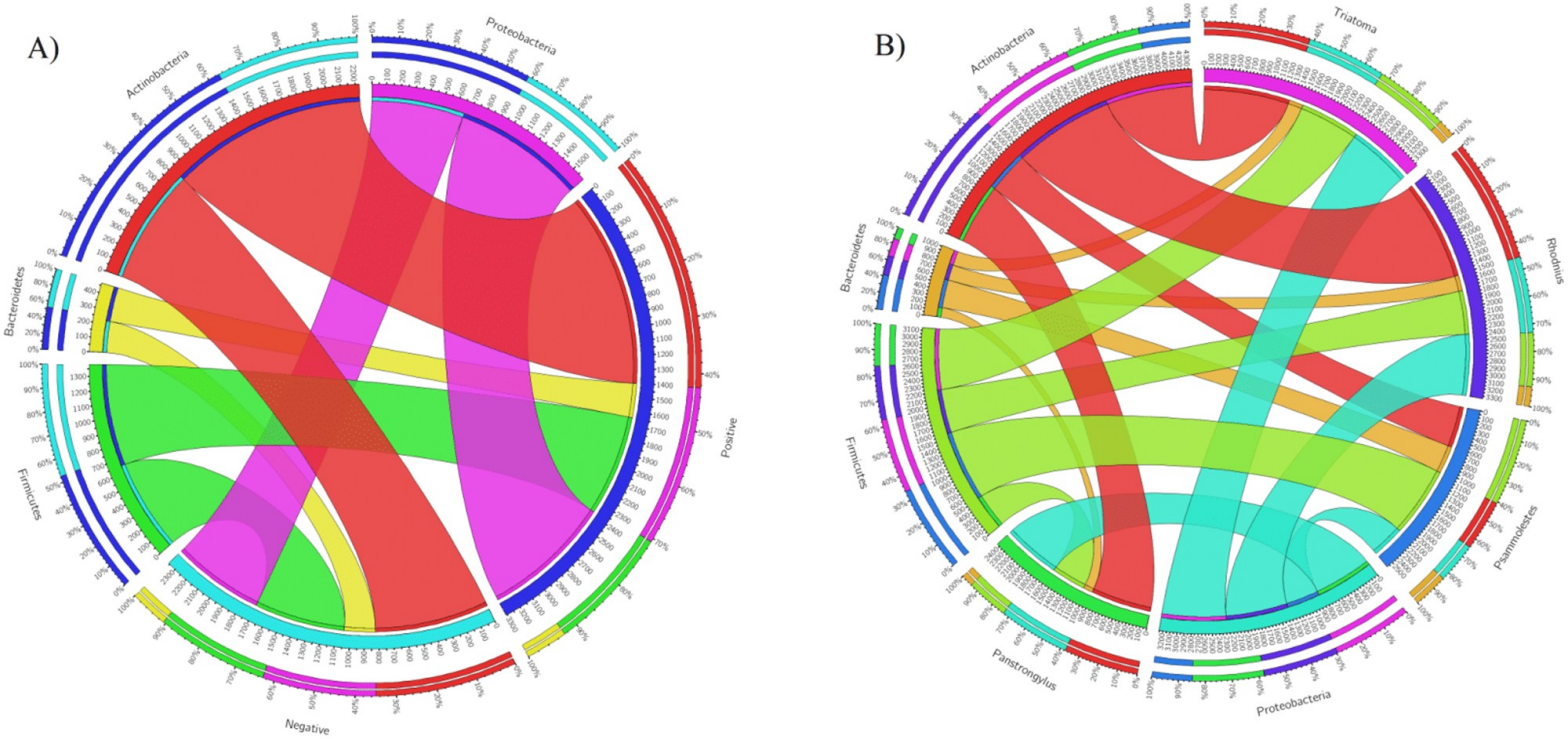
Circos plots. (A) Observing each main bacterial phyla and *T*. *cruzi* absence/presence. (B) Observing each main bacterial phyla and each triatomine species. Outer bars show the percentage of reads in a category that are connected to the category at the other end of the drawn band.

The composition of bacterial phyla in the gut of the insects was significantly different between triatomine species (χ^2^ = 133130, df = 1134, *p* < 0.0001), whereas there was no significant difference between the state of *T*. *cruzi* infection. Circos plots showed that *T*. *cruzi*-positive samples presented a higher association with Actinobacteria (approximately 40%) and Proteobacteria (approximately 30%), while the association with Bacteroidetes (less than 10%) and Firmicutes (approximately 20%) appeared to be less clear (Fig 2A). Additionally, Actinobacteria seemed to be more abundant in *Rhodnius*, Bacteroidetes and Firmicutes were slightly more abundant in *Ps*. *arthuri*, and Proteobacteria was more abundant in *Triatoma* and *P*. *geniculatus* (Fig 2B). Despite these being the most evident associations, there were differences in the abundance of all of the phyla between *T*. *cruzi* positive and negative samples.

The abundance of bacterial phyla was also compared between DTUs, with Actinobacteria most abundant in TcII-TcVI samples (58.4%), while Proteobacteria was the most abundant phylum in TcI samples (35.5%). Firmicutes abundance remained constant independent of the DTU, but changes in the other constituents of the gut bacterial communities were dependent on the DTU (S7 Fig).

### Diversity analysis

**Fig 3:**
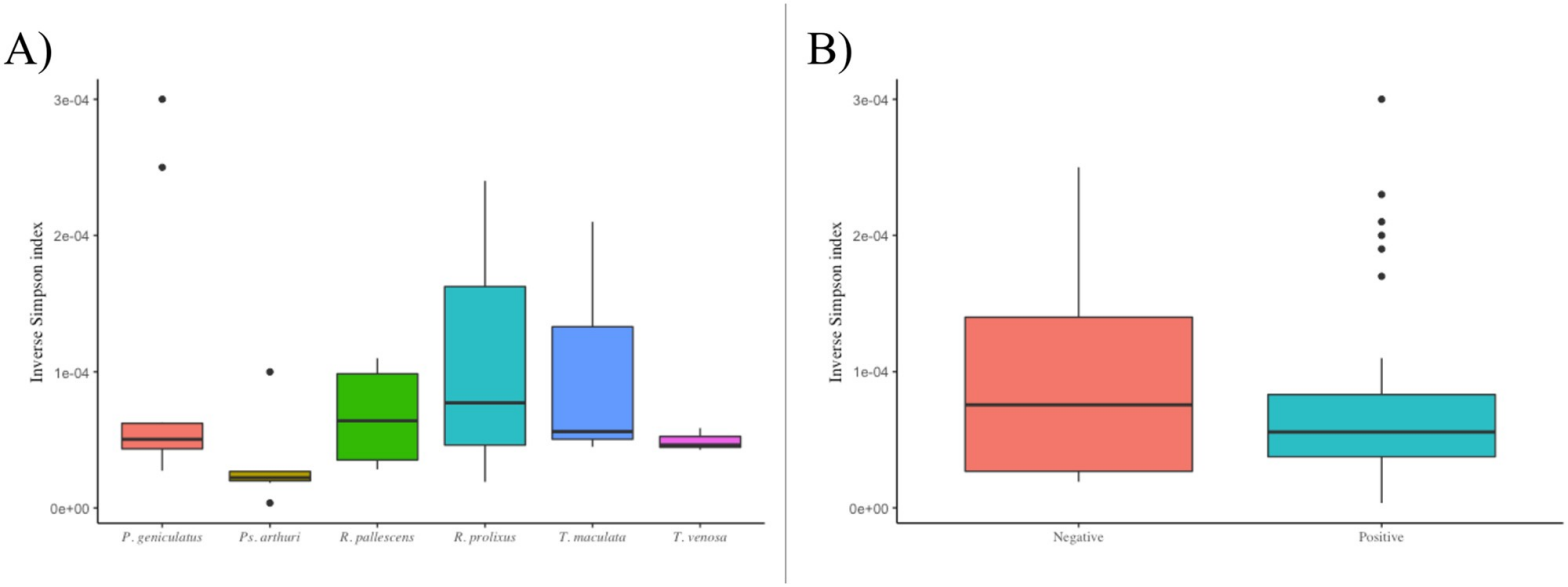
Inverse Simpson index for (A) each triatomine species and (B) presence/absence of *T*. *cruzi*.

For alpha diversity analysis, we calculated the inverse Simpson diversity index for the data separated by triatomine species and *T*. *cruzi* infection. The highest and lowest values were calculated for *P*. *geniculatus* and *Ps*. *arthuri*, respectively (Fig 3A). In the case of *T*. *cruzi* infection, the inverse Simpson index was predominantly higher in negative samples (negative average = 1.010 × 10^˗4^; positive average = 7.628 × 10^˗5^) (Fig 3B). The overall Margalef richness index was 0.8167, although this value varied for each triatomine species and the state of infection (see S8 Fig). For beta diversity analysis, we calculated the Bray-Curtis dissimilarity for the gut bacterial composition by triatomine species and department. The highest values were seen when Guajira was compared with the other departments, which suggests that the bacterial species found in the triatomines belonging to this department were least similar to bacteria found in the other departments (see S9 Fig). As for the ecotopes, we found the highest value of Bray-Curtis dissimilarity between Domestic and Sylvatic, which suggests that there is an apparent difference between the bacterial species found in triatomines that belong to these ecotopes (see S9 Fig).

**Fig 4:**
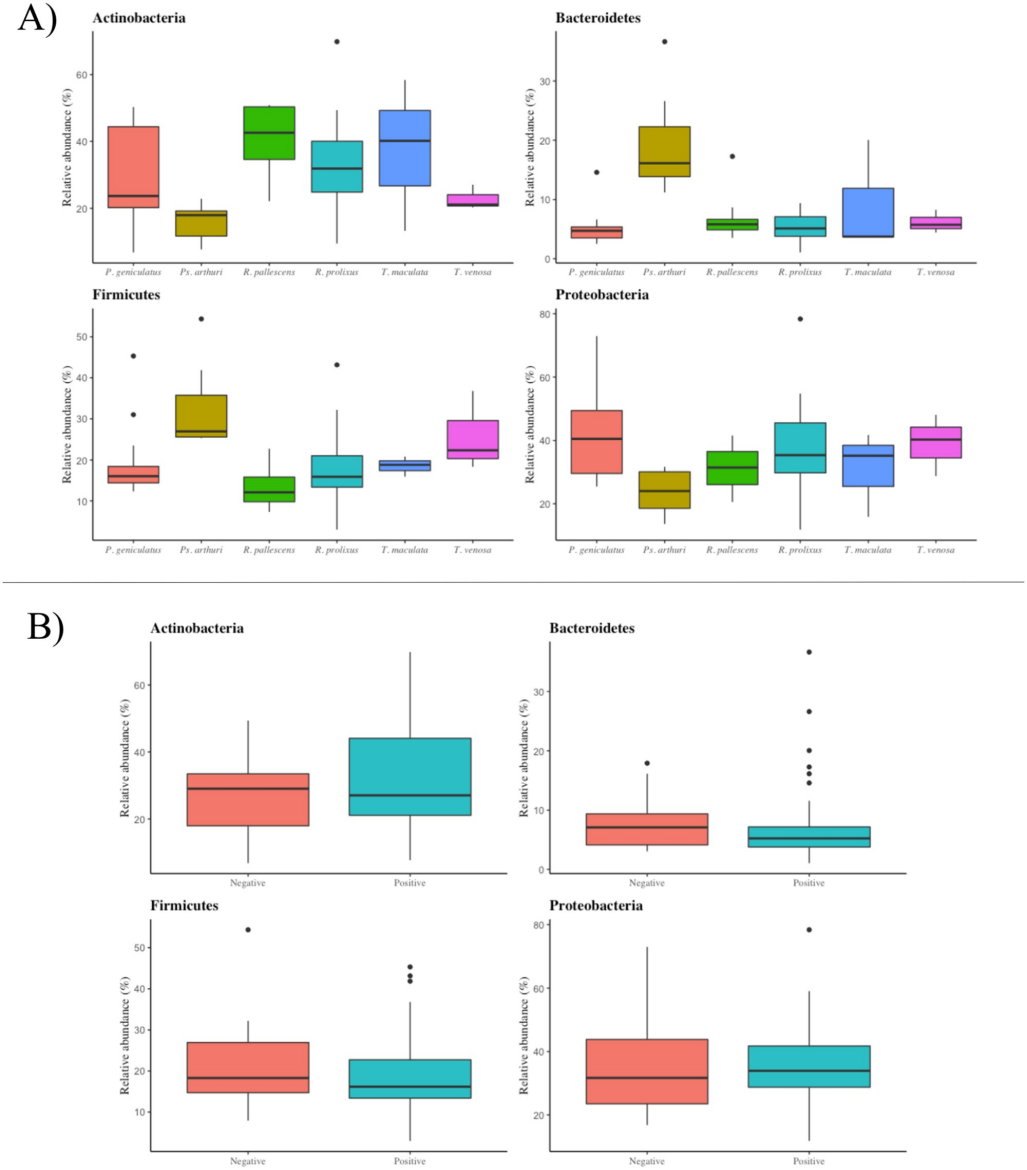
Bray-Curtis dissimilarity. The two “sites” grouping used for this test were the departments where the samples were collected (A) and the ecotopes observed surrounding the samples when they were collected (B).

Additionally, we compared these indexes for the main phyla found in each triatomine species and *T*. *cruzi* presence/absence. For triatomine species, Bacteroidetes and Firmicutes had a higher presence in *Ps*. *arthuri* than other triatomines, while Proteobacteria was more abundant in *P*. *geniculatus* than in the other species. For Actinobacteria, its presence reached 40% in every triatomine species except *Ps*. *arthuri* and *T*. *venosa* (Fig 4A). For *T*. *cruzi* presence, Actinobacteria seemed to be more abundant in positive samples, while the reverse was observed for Proteobacteria (Fig 4B).

## Discussion

The understanding of the microbiota inhabiting vectors of human-borne diseases is pivotal for the comprehension of environment and ecological interactions that frame maturation, colonization, and subsequent transmission of high impact parasites. To our knowledge, this is the first study that has used the main *T*. *cruzi* vector species in South America for assessment of their gut bacterial structure applying next generation sequencing (NGS) technologies. Our results suggest that NGS technologies can be used to detect a big part of the diversity harbored in insect guts and that these technologies can have a crucial role in the understanding of the ecology of Chagas disease, especially the transmission dynamics of the parasite.

In this study, we found 27 bacterial phyla (Fig 1, S2 Fig), indicating higher diversity than in previous studies [18,20,21,36]. This may be a consequence of the methodology used, which had greater sensitivity (higher number of reads) and used primers that could be more informative regarding the hypervariable V4 region of the 16S rRNA gene, conditions that can influence detection [37]. Nonetheless, other studies have also found the four predominant phyla in the gut of triatomines that we found in this study: Actinobacteria, Bacteroidetes, Firmicutes, and Proteobacteria [20,21,38]. There were no significant differences between positive and negative samples for *T*. *cruzi* presence, but each species showed a different bacterial signature (Fig 1, S6 Fig), which confirms that bacterial abundance is species-dependent, as previous studies have suggested [6]. Our study indicates that insects without *T*. *cruzi* had higher bacterial diversity (Fig 3), although there was no statistical difference. In previous studies, bacterial diversity tended to increase with *T*. *cruzi* infection [17,22], which was proposed as a defense mechanism of parasite immune modulation [6]. However, some studies have found that *T*. *cruzi* immune response modulation decreases gut microbiota given that this can trigger antibacterial activity [14,39]. It should also be taken into account that, unlike previous studies [20,21], the samples used in this study were field-collected, which can influence the previously reported apparent effect of *T*. *cruzi* in the bacterial diversity [40]. Given that Circos plots did not show a difference between *T*. *cruzi* positive and negative states (Fig 2A), we suggest that bacterial communities composition could be independent of the parasite presence, although further analysis is needed to confirm this. Previous work has already suggested that only some bacteria possess mechanisms to recognize *T*. *cruzi* upon its entrance and, consequently, react to trigger the immune response [41]. Despite all this, our study is limited by the sample size, therefore, future studies are needed to clarify this topic, using more insects that come from more geographic regions. Furthermore, our methodology does not allow to identify the exact section of the gut where *T*. *cruzi* is present, therefore, it remains a possibility that the parasite transiently affects the bacterial communities when it is located in certain regions of the gut. In the future, all this could be addressed by performing more precise dissections of the insects and mechanistic experiments in which the functionality of the bacteria is considered as the focus of the study, as has been proposed for *Leishmania* and *Lutzomyia* [42]. Additionally, geographic location was ultimately not included in the main analyses of our study given the differences in sample size, and this aspect should be improved in upcoming studies.

Despite statistically significant differences between some bacterial phyla (Actinobacteria-Firmicutes, *p* = 0.0109; Firmicutes-Proteobacteria, *p* = 0.0001), there are common trends between the gut bacteria of different triatomine species, like the presence of the four predominant phyla previously mentioned. Actinobacteria possess a wide range of physiological and metabolic properties, such as the formation of secondary metabolites and antimicrobial bioactive compounds [43]. Proteobacteria are tightly related to dysbiosis in vertebrates and harbors a great part of the functional variation in contrast with other highly abundant phyla like Bacteroidetes or Firmicutes [44,45]. Bacteroidetes, commonly found in the gut microbiota of animals, are known for their ability to degrade polysaccharides due to numerous carbohydrate-active enzymes [46,47]. Firmicutes include several genera that may help to salvage energy from unabsorbed dietary carbohydrate given their carbohydrate fermentation abilities [48]. Additionally, Actinobacteria and Firmicutes are thought to supplement nutrition and are required for normal growth of insects [37,49,50]. Some bacteria belonging to Bacteroidetes can degrade porphyrans and polysaccharides, enabling degradation of food components [51]. Proteobacteria can be important for digestion, immunity, and reproduction in insects [50,52–55]. In our study, these four bacterial phyla showed the highest abundances, which we can relate to the assistance they provide to the physiological processes mentioned, making them fundamental to the insect and the vertebrates it feeds on.

Regarding the bacterial genera and species present, several are worthy of mention. *Wolbachia* was only found in *R*. *prolixus* (S4 Fig, S2 Table), while it was also found in *R*. *pallescens* in previous studies [20,21,35]. Given that these bacteria can alter the reproductive tissue of their host, they have been suggested as a tool for vector control in *Aedes* [35,56] and *Lutzomyia* [57,58]; this may be the reason why, in this study, they were only detected at low abundance, in one triatomine species, and in a bug that tested negative for the presence of the parasite (see S2 Table). To further elucidate the colonization profile of these specific bacteria in triatomines, it will be essential to perform further studies where a greater number of parasites are sampled, the provenance regions of samples are more diverse, and more specific tests are carried out. There are also differences between our findings to those of previous studies regarding the presence of some bacteria in certain triatomine species [22,38]. These differences suggest that the presence of certain bacteria may depend on other, as yet uncharacterized, variables and further studies are therefore needed. Additionally, there were some individual results that were worth noting but were not fully explainable; further studies may be necessary to fully understand them. For example, we found *Cyanobacteria* in some triatomine guts. These bacteria have not previously been associated with the gut microbiota of triatomines or any other insect [37,40] but similar bacteria have been found in the human gut [59] and are therefore worth further investigation. We also found *Tenericutes* in the triatomine gut and, given that this type of bacteria can be commensal or parasitic in animals [60], its presence may potentially be related to the feeding source of the triatomine, although this requires verification. *Serratia* and *Rhodococcus* were also detected at low abundances in our study (S4 Fig, S2 Table). Species belonging to these genera have been previously reported as being affected by the presence of trypanosomatids [39,61], and, particularly for *S*. *marcescens*, a possible trypanolytic effect of this bacterial species has been suggested; however, both effects are apparently dependent on the strain of *T*. *cruzi* present [34]. Given that both *Serratia* and *Rhodococcus* were found in this study, it would be valuable to thoroughly check these species in further studies to identify the bacterial species present and their potential effect on the parasite. Furthermore, it would be valuable to isolate these bacteria and evaluate the production of secondary metabolites *in vitro* that could negatively affect the establishment of *T*. *cruzi* in the triatomine gut, especially considering that some of these bacteria have been previously suggested as producers of trypanolytic factors [34].

We also found that the abundance of reads belonging to each phylum varied among the triatomine species evaluated in this study (Fig 4A, S8 Fig). Although there are general similarities, an apparent difference can be seen between *Ps*. *arthuri* and the rest of triatomine species, which is also suggested by the PCA (S10 Fig). Despite Bacteroidetes being the least abundant of the four main phyla, *Ps*. *arthuri* individuals had the most Bacteroidetes reads compared with the other triatomines. Likewise, Firmicutes, the second least abundant of the main phyla, was also most abundant in *Ps*. *arthuri* individuals. A similar phenomenon can be observed between Actinobacteria and *Ps*. *arthuri*: despite being one of the most abundant phyla, *Ps*. *arthuri* individuals tended to have fewer of these reads than the rest of triatomine species. This could suggest that *Ps*. *arthuri* has a different gut bacterial signature than the rest of the triatomine species evaluated in this study, which could be explained by the specialization in *Ps*. *arthuri* feeding habits, where they exploit bird nests instead of feeding on a wide range of mammals, like the other triatomine species [62]. Exploring these characteristics of *Ps*. *arthuri* could be worthy of future studies, especially given the recent evidence of *T*. *cruzi* infection in this species [63,64]. Moreover, the PCA results suggest that gut bacterial signatures could exist not only in *Ps*. *arthuri* but in other triatomine species, although in our study there was little clustering of samples based on species identity and overlaps were observed (S10 Fig).

## Conclusions

We were able to find a higher diversity than reported previously for bacterial taxa present in the triatomine gut, possibly as a result of the methodology used. Gut bacterial structure seems to vary in a species-dependent manner. According to our results, ecotope, geographic location, and feeding habits [65] might be variables that need to be studied in more depth as sources of variation. This study allowed us to characterize the gut bacterial structure of the main triatomine genera in Colombia and more studies should be made to completely understand the ecology and role of triatomine gut microbiome interacting with *T*. *cruzi*, which might provide new insights regarding host-pathogen interactions and subsequently the continental ecology of Chagas disease.

## Supporting information

S1 Fig Geographical distribution of the 54 triatomine samples used. Legends indicate each triatomine genus collected and the figure size indicates the triatomine density in the location.
(PDF)

S2 Fig Relative abundance of bacterial phyla in each triatomine. (PDF)

S3 Fig Rarefaction curves of 16S rRNA of individual triatomines. The triatomines analyzed belonged to (A) *P*. *geniculatus*, (B) *Ps*. *arthuri*, (C) *Rhodnius* (*R*. *pallescens* and *R*. *prolixus*) and (D) *Triatoma* (*T*. *maculata* and *T*. *venosa*). Continuous lines represent the *T*. *cruzi*-positive individuals and dotted lines represent the *T*. *cruzi*-negative samples.
(PNG)

S4 Fig Relative abundance of bacterial genera from the main bacterial phyla per individual. (PDF)

S5 Fig Abundance of relevant bacterial genera in each triatomine species. (PDF)

S6 Fig Statistically significant differences between bacterial phyla abundance in each triatomine species. Each cell with a red color represents a comparison where there was a statistically significant (*p* < 0.05) difference in a previously performed t-test, while the blank cells indicate a lack of statistical difference for the comparison. Gray cells do not contain any information because the comparison was not possible (same triatomine species) or the comparison has already been considered in the table.
(PDF)

S7 Fig Relative abundance of bacterial phyla in triatomines with different *T*. *cruzi* DTUs. (PDF)

S8 Fig Margalef index for bacteriome data. Data are presented according to (A) triatomine species and (B) *T*. *cruzi* infection.
(PNG)

S9 Fig Individual relative abundance of each main bacterial phyla (A) in each triatomine species, and (B) depending on the presence or absence of *T*. *cruzi*. (PNG)

S10 Fig Principal component analysis (PCA) of the number of reads corresponding to each main bacterial phylum. Each triatomine sample is represented by a dot. The first two components accounted for 52.7% of variance.
(PNG)

S1 Table Complete dataset of triatomines used. This dataset contains information regarding *T*. *cruzi* presence and DTU information, geographic location with the corresponding coordinates, ecotope, and sex of the triatomine.
(XLSX)

S2 Table Complete dataset of read counts for the main bacterial phyla and their corresponding genera. This dataset also shows each triatomine individual to whom the reads belong, along with its species and if *T*. *cruzi* was present or absent.
(XLSX)
