# Supplementary figures and images for: Species-dependent variation of the gut bacterial communities across Trypanosoma cruzi insect vectors
Source: PLoS One. 2020 Nov 12;15(11):e0240916. doi: 10.1371/journal.pone.0240916 (PMC7660481; doi:10.1371/journal.pone.0240916)

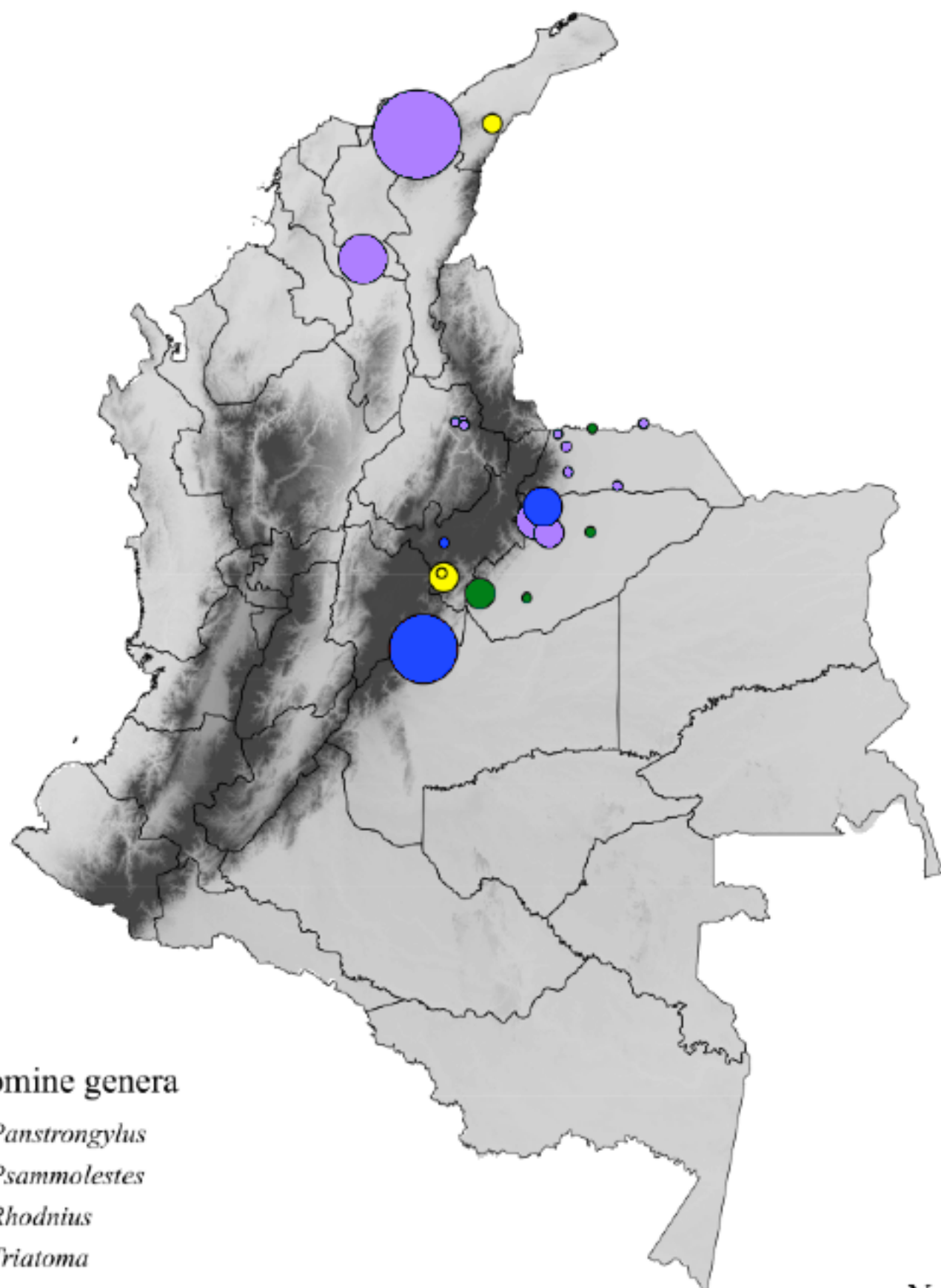

Triatomine genera

- *Panstrongylus*
- *Psammolestes*
- *Rhodnius*
- *Triatoma*

0 62,5 125 250 375 500  
Kilometers

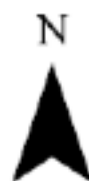

Supplement: S1 Fig — Legends indicate each triatomine genus collected and the figure size indicates the triatomine density in the location. (PDF) [file pone.0240916.s001.pdf]

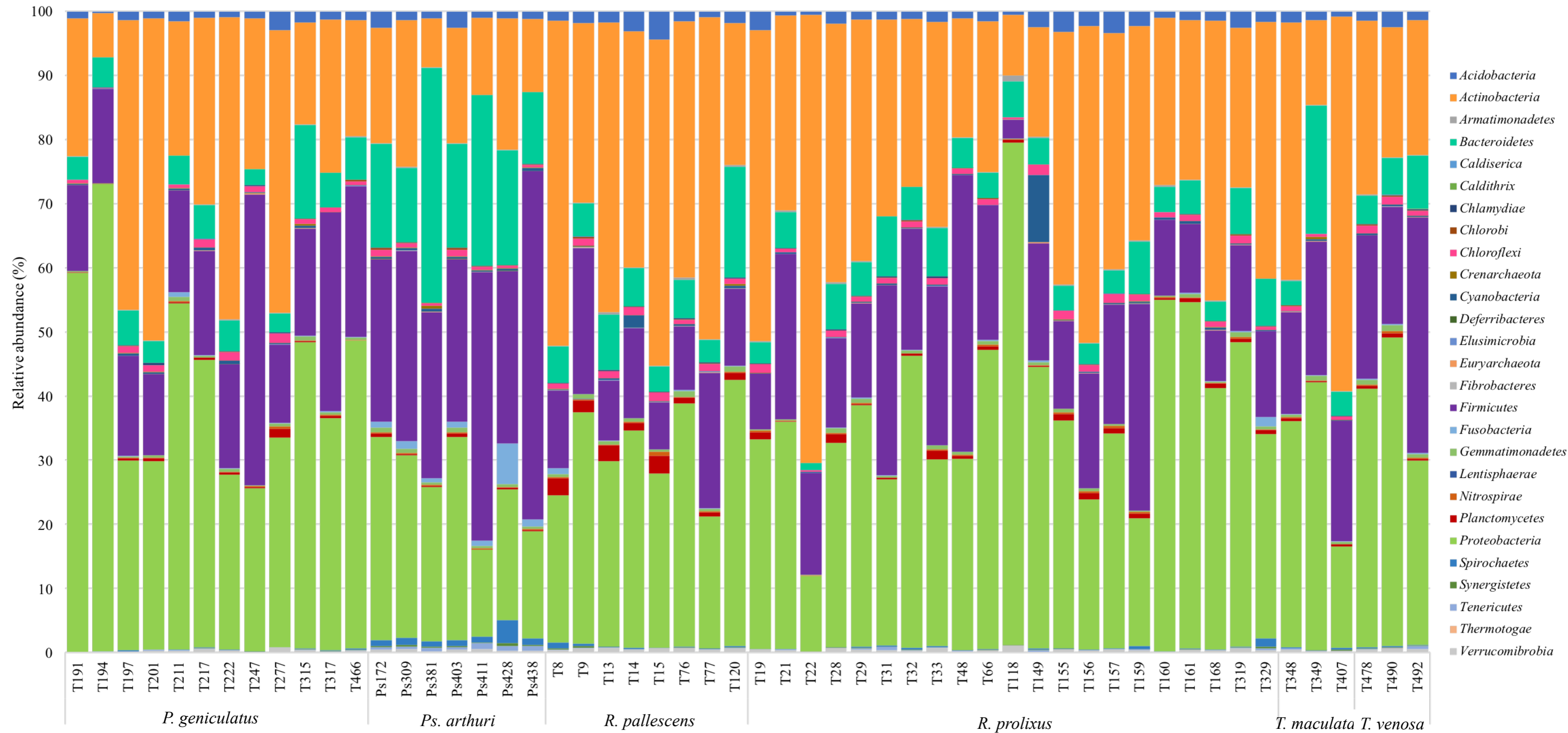

Supplement: S2 Fig — (PDF) [file pone.0240916.s002.pdf]

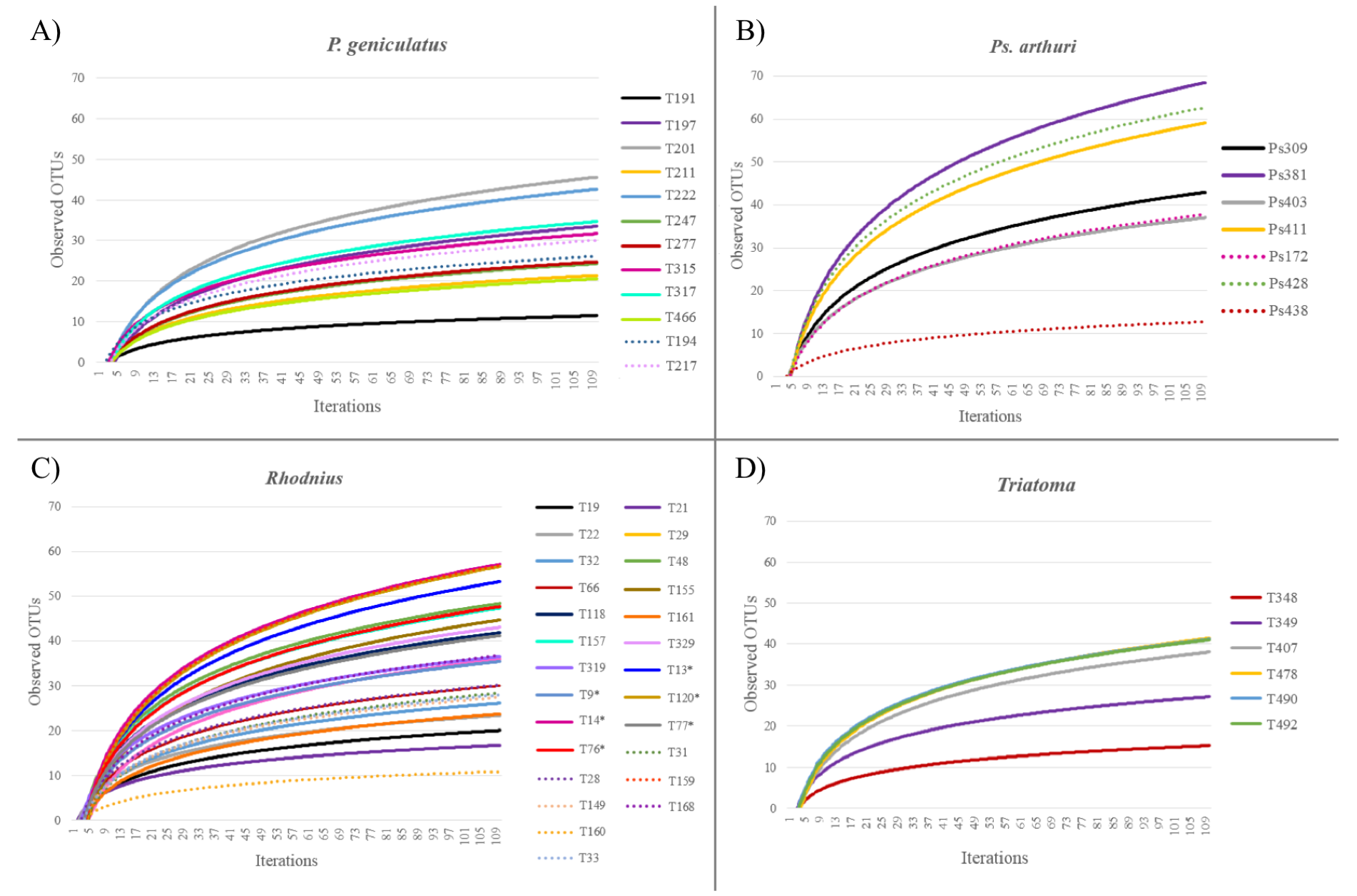

Supplement: S3 Fig — The triatomines analyzed belonged to (A) P. geniculatus, (B) Ps. arthuri, (C) Rhodnius (R. pallescens and R. prolixus) and (D) Triatoma (T. maculata and T. venosa). Continuous lines represent the T. cruzi-positive individuals and dotted lines represent the T. cruzi-negative samples. (PNG) [file pone.0240916.s003.png]

# Actinobacteria

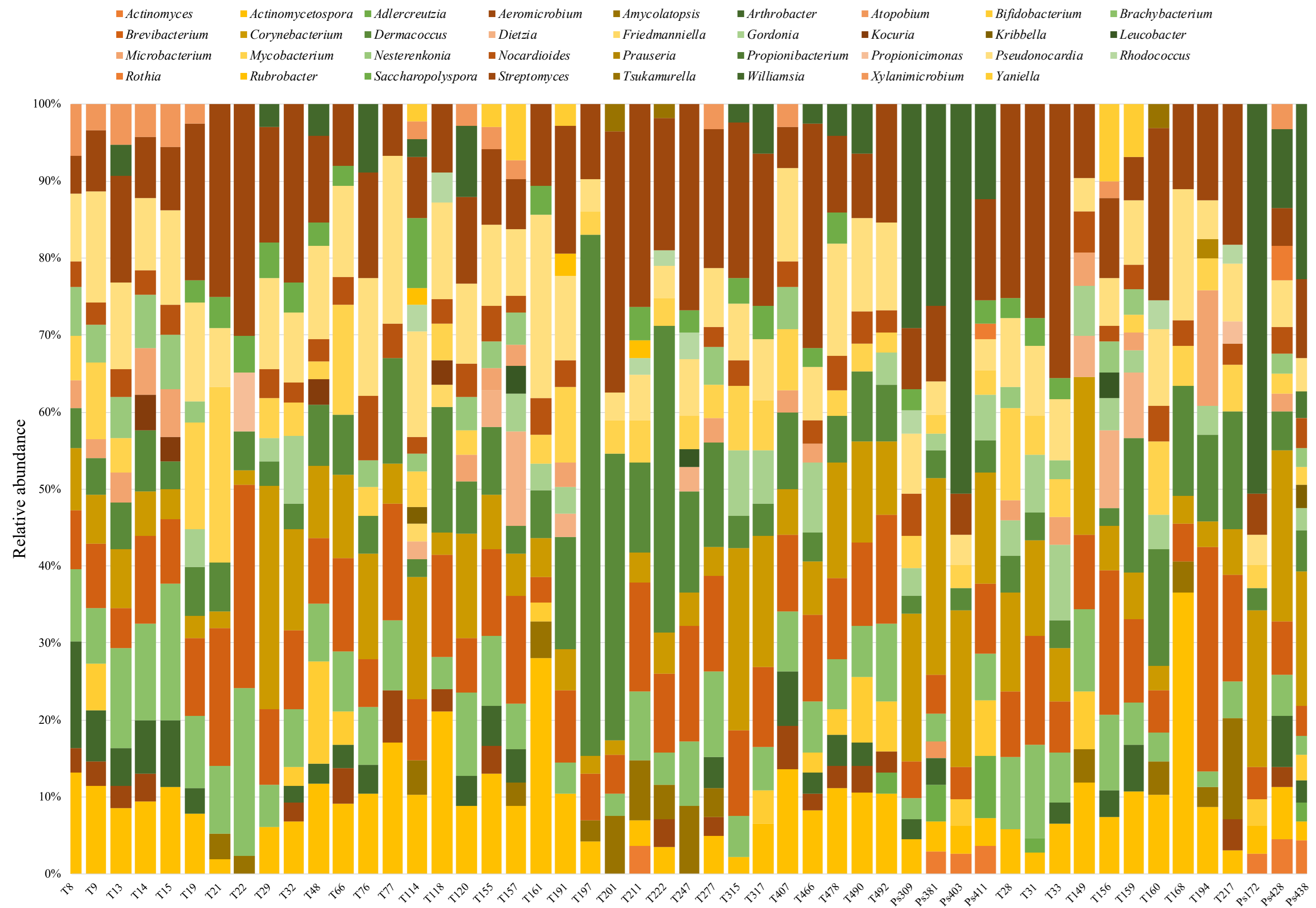

# Bacteroidetes

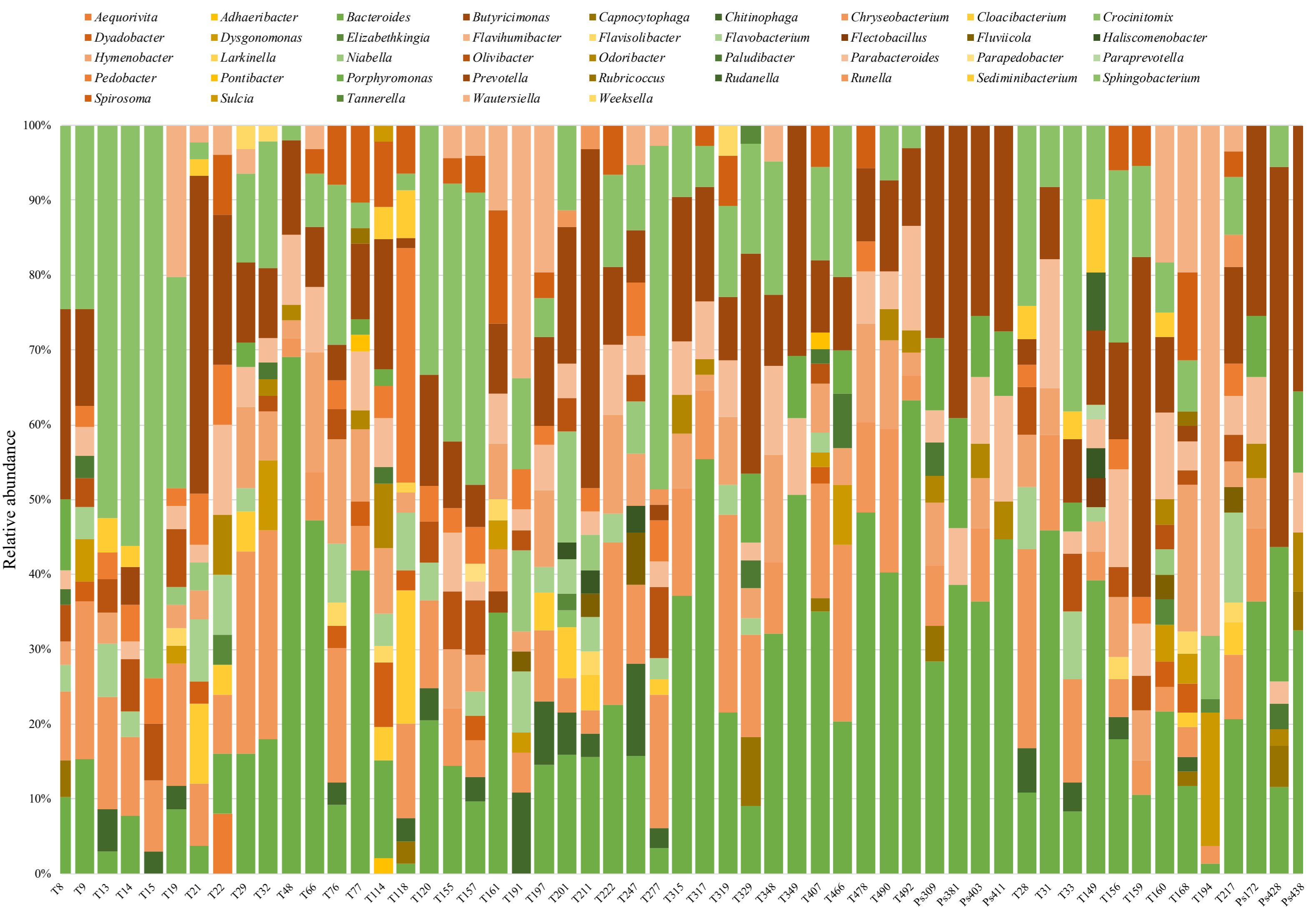

# Firmicutes

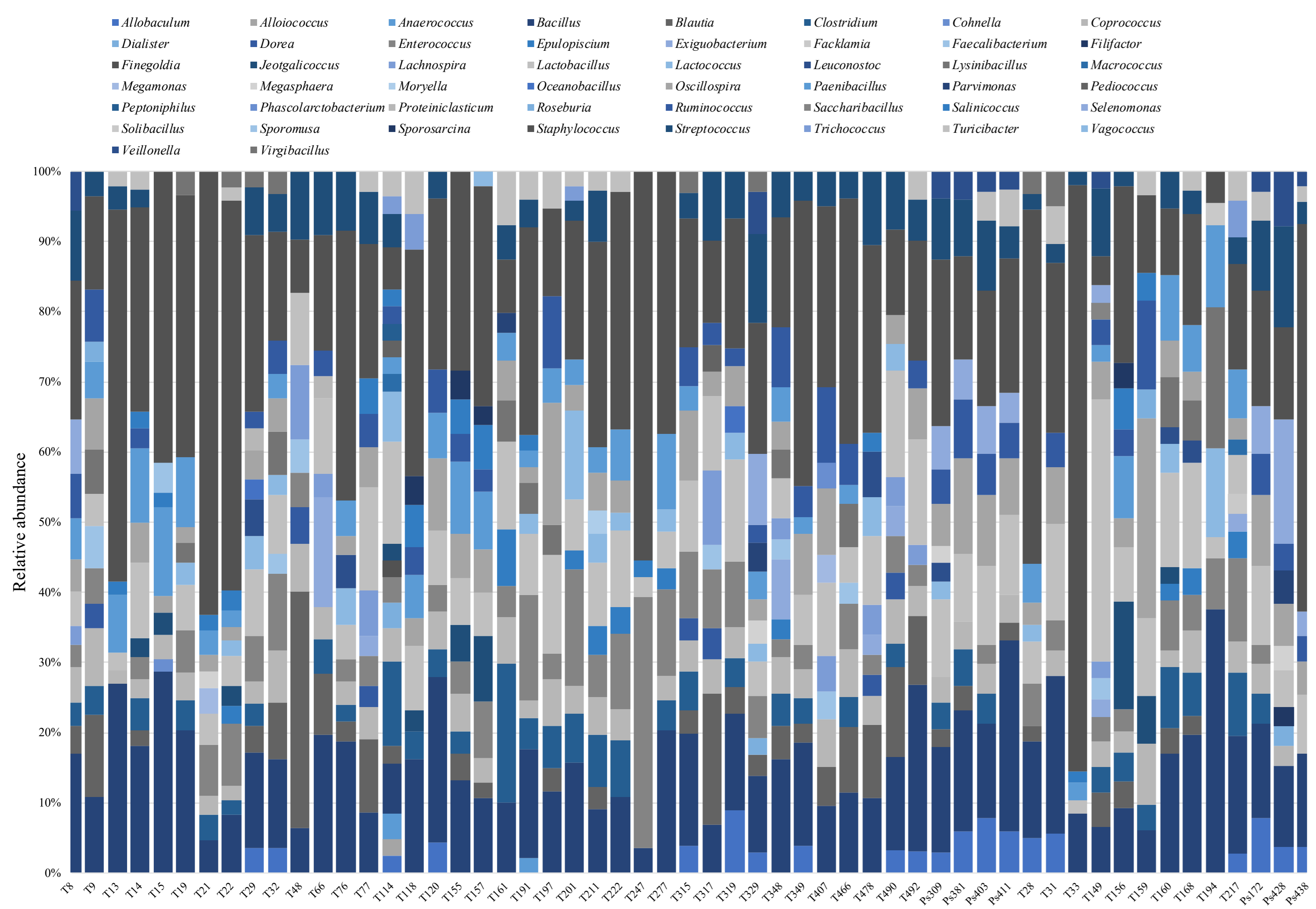

# Proteobacteria

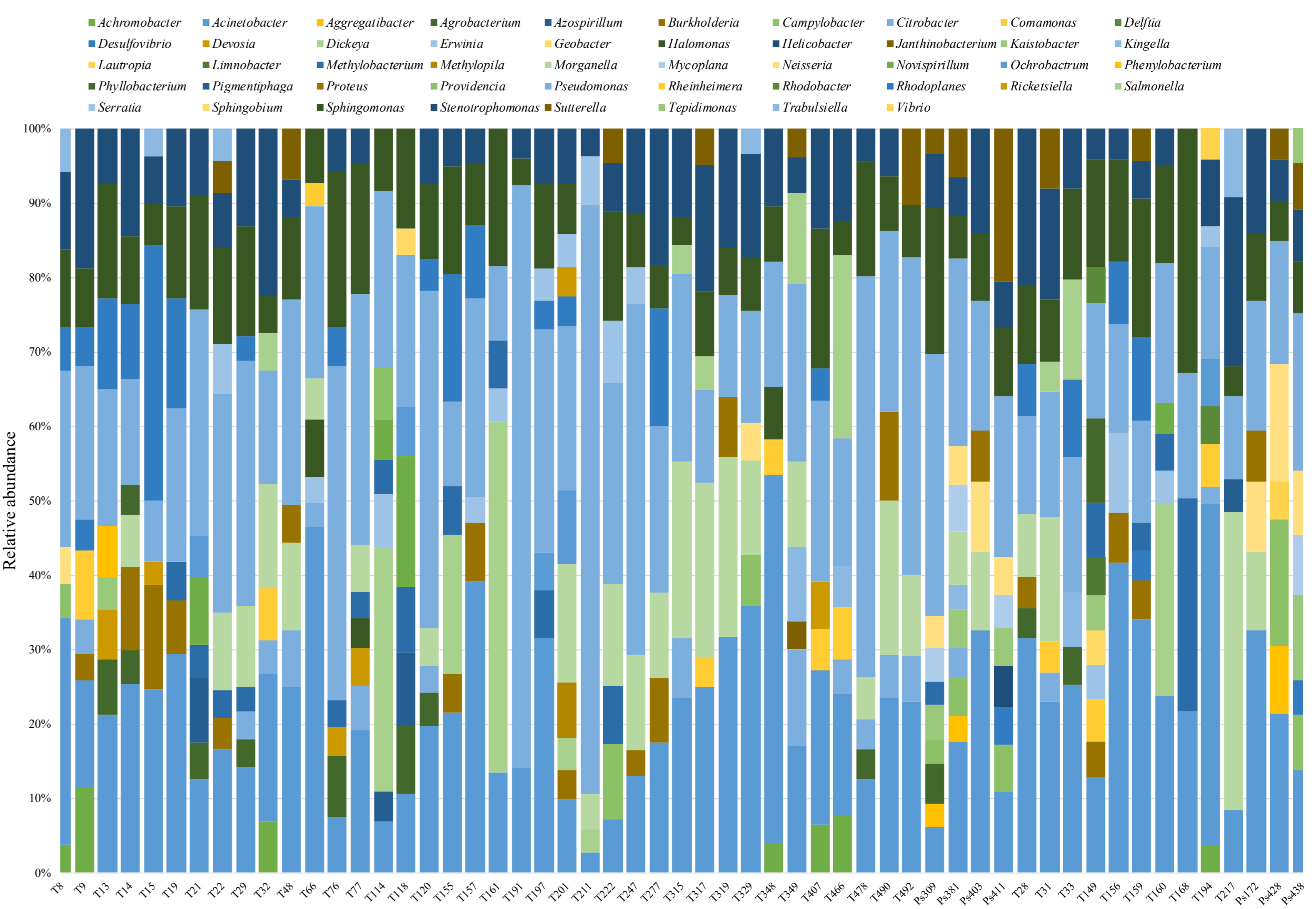

Supplement: S4 Fig — (PDF) [file pone.0240916.s004.pdf]

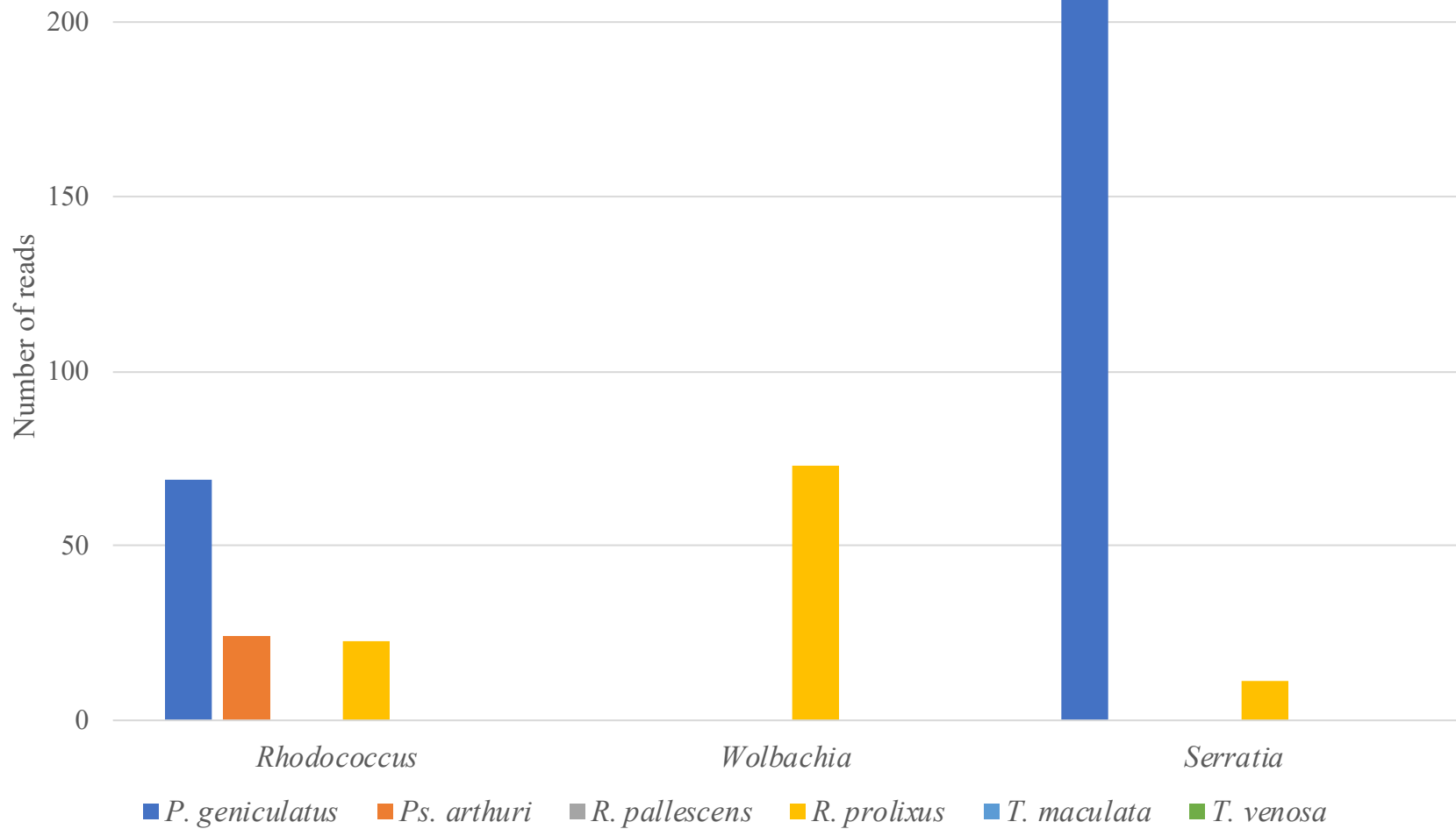

Supplement: S5 Fig — (PDF) [file pone.0240916.s005.pdf]

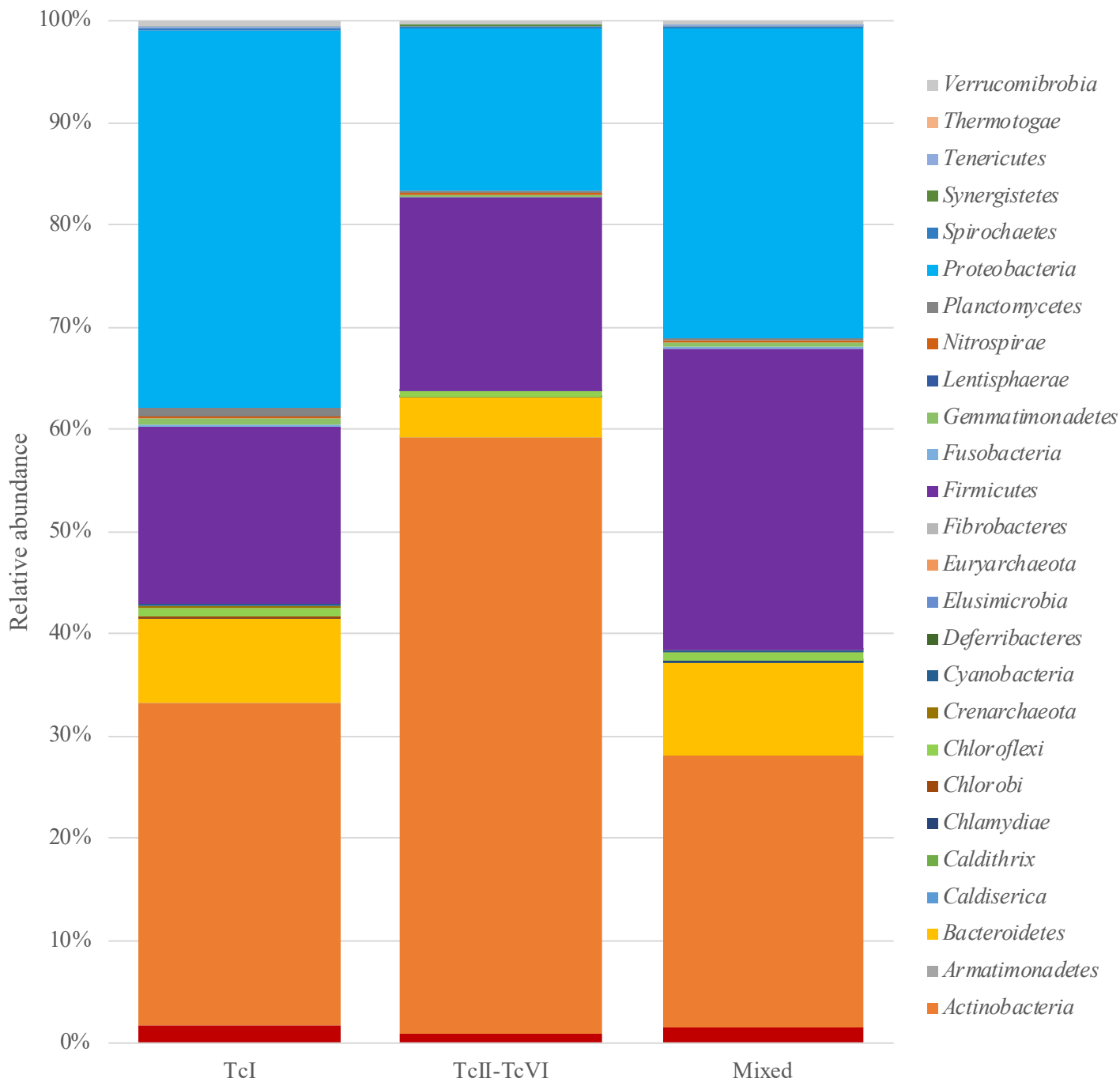

Supplement: S7 Fig — (PDF) [file pone.0240916.s007.pdf]

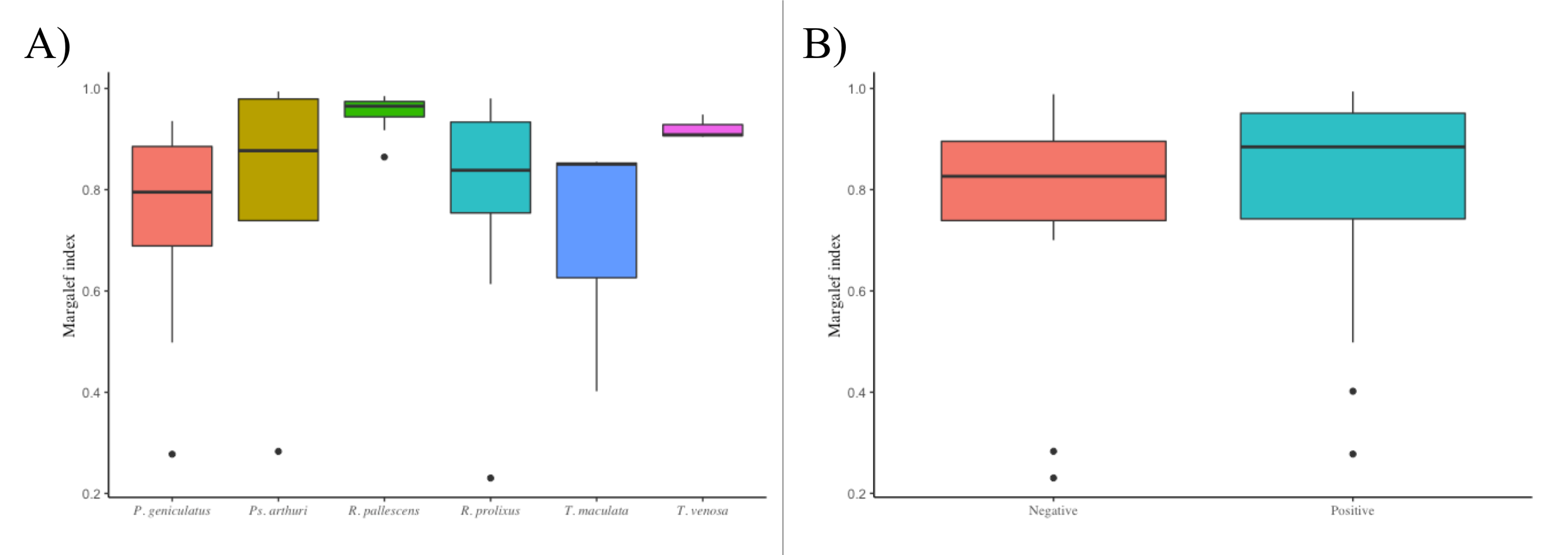

Supplement: S8 Fig — Data are presented according to (A) triatomine species and (B) T. cruzi infection. (PNG) [file pone.0240916.s008.png]

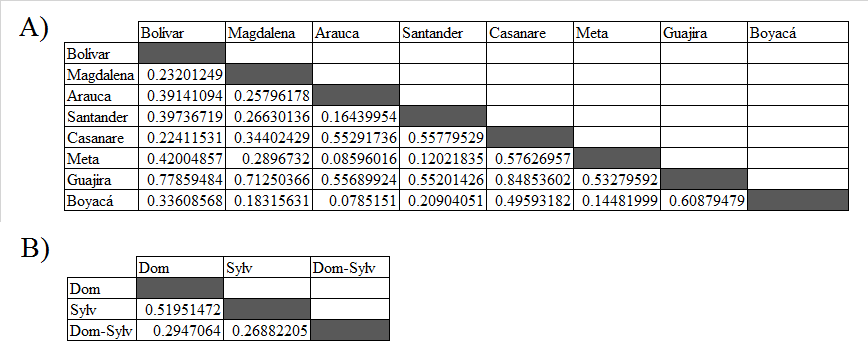

Supplement: S9 Fig — (PNG) [file pone.0240916.s009.png]

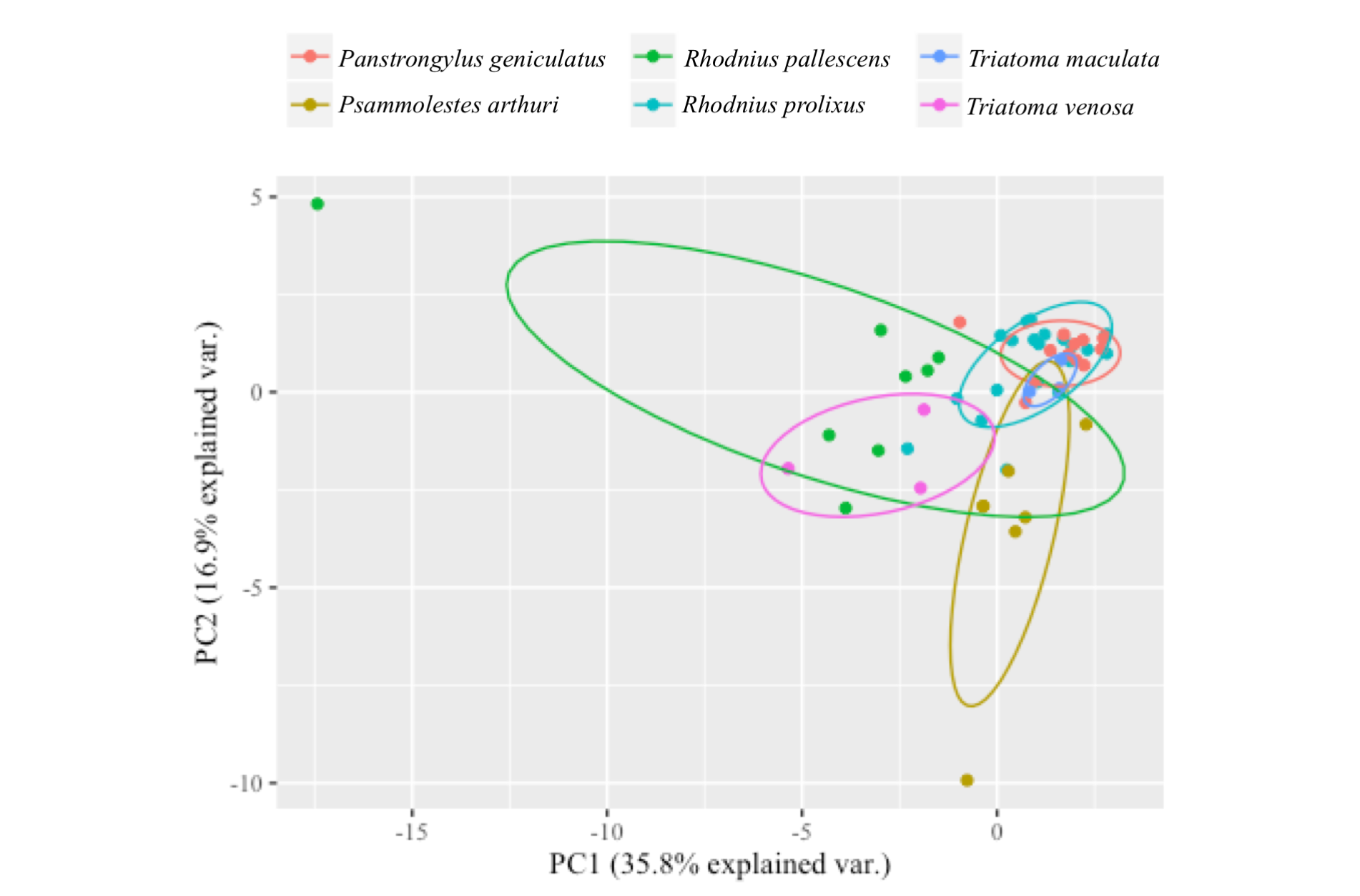

Supplement: S10 Fig — Each triatomine sample is represented by a dot. The first two components accounted for 52.7% of variance. (PNG) [file pone.0240916.s010.png]
