# Supplementary material for: Species-dependent variation of the gut bacterial communities across Trypanosoma cruzi insect vectors
Source: PLoS One. 2020 Nov 12;15(11):e0240916. doi: 10.1371/journal.pone.0240916 (PMC7660481; doi:10.1371/journal.pone.0240916)
Supplement: S6 Fig — Each cell with a red color represents a comparison where there was a statistically significant (p < 0.05) difference in a previously performed t-test, while the blank cells indicate a lack of statistical difference for the comparison. Gray cells do not contain any information because the comparison was not possible (same triatomine species) or the comparison has already been considered in the table. (PDF) [file pone.0240916.s006.pdf]

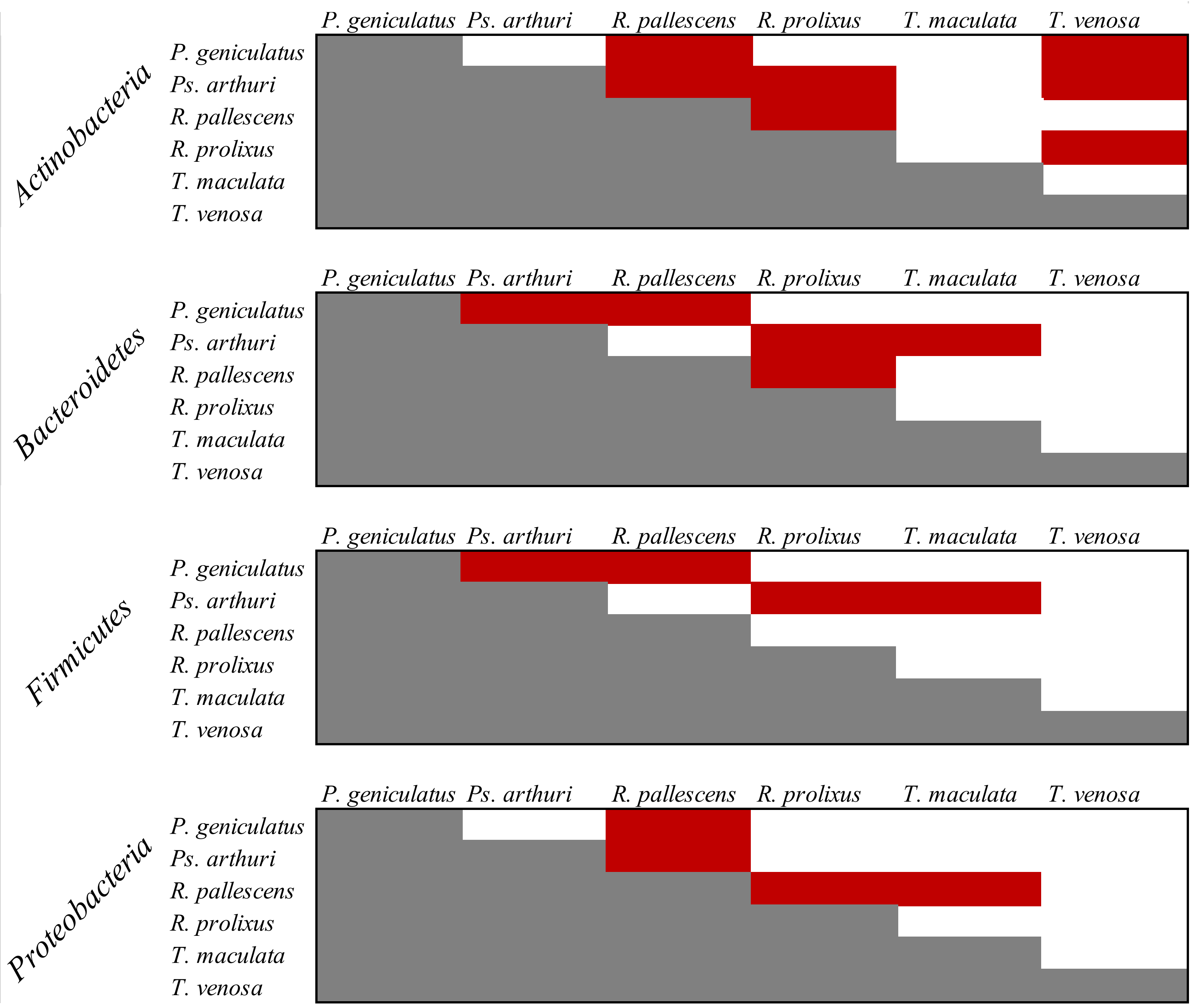
Actinobacteria
Bacteroidetes
Firmicutes
Proteobacteria
P. geniculatus
Ps. arthuri
R. pallescens
R. prolixus
T. maculata
T. venosa
